# DI2: prior-free and multi-item discretization of biological data and its applications

**DOI:** 10.1186/s12859-021-04329-8

**Published:** 2021-09-08

**Authors:** Leonardo Alexandre, Rafael S. Costa, Rui Henriques

**Affiliations:** 1 IDMEC, Instituto Superior Técnico, Universidade de Lisboa, Av. Rovisco Pais, 1049-001 Lisbon, Portugal; 2 INESC-ID, Lisbon, Portugal; 3 Instituto Superior Técnico, Universidade de Lisboa, Lisbon, Portugal; 4 LAQV-REQUIMTE, DQ, NOVA School of Science and Technology, Universidade NOVA de Lisboa, 2829-516 Caparica, Portugal

**Keywords:** Multi-item discretization, Prior-free discretization, Heterogeneous biological data, Data mining

## Abstract

**Background:**

A considerable number of data mining approaches for biomedical data analysis, including state-of-the-art associative models, require a form of data discretization. Although diverse discretization approaches have been proposed, they generally work under a strict set of statistical assumptions which are arguably insufficient to handle the diversity and heterogeneity of clinical and molecular variables within a given dataset. In addition, although an increasing number of symbolic approaches in bioinformatics are able to assign multiple items to values occurring near discretization boundaries for superior robustness, there are no reference principles on how to perform multi-item discretizations.

**Results:**

In this study, an unsupervised discretization method, DI2, for variables with arbitrarily skewed distributions is proposed. Statistical tests applied to assess differences in performance confirm that DI2 generally outperforms well-established discretizations methods with statistical significance. Within classification tasks, DI2 displays either competitive or superior levels of predictive accuracy, particularly delineate for classifiers able to accommodate border values.

**Conclusions:**

This work proposes a new unsupervised method for data discretization, DI2, that takes into account the underlying data regularities, the presence of outlier values disrupting expected regularities, as well as the relevance of border values. DI2 is available at https://github.com/JupitersMight/DI2

**Supplementary Information:**

The online version contains supplementary material available at 10.1186/s12859-021-04329-8.

## Background

Approaches to discretization of continuous variables have long been discussed alongside their pros and cons. Altman et al. [1] and Bennette et al. [2] both discuss the relevance and impact of categorizing continuous variables and reducing the cardinality of categorical variables. Liao et al. [3] compares various categorization techniques in the context of classification tasks in medical domains, without using domain knowledge of field experts. Considerable advances in data mining are being driven by symbolic approaches, particularly those rooted in bioinformatic, compression and pattern mining research, including contributions pertaining to the analysis of symbolic sequences, text or basket transactions. The relevance of discretization meets both descriptive and predictive ends, encompassing state-of-the-art approaches such as pattern-based biclustering [4] and associative models such as XGBoost [5].

In this work we present DI2, a Python library that extends non-parametric tests to find the best fitting distribution for a given variable and discretize it accordingly. DI2 offers three major contributions: (i) corrections to the empirical distribution before statistical fitting to guarantee a more robust approximation of candidate distributions; (ii) efficient statistical fitting of 100 theoretical probability distributions; and, finally, (iii) assignment of multiple items according to the proximity of values to the boundaries of discretization, a possibility supported by numerous symbolic approaches [4, 6, 7]. The assignment of multiple items [8], generally referred as multi-item discretization, conferes the possibility to avail the wealth of data structures and algorithms from the text processing and bioinformatics communities without the risks of the well-studied item-boundaries problem.

Discretization methods have wide taxonomy [9] with a determinant division in: (1) supervised, where the method uses the class variable to bin the data, and, (2) unsupervised, where the method is independent of the class variable. DI2 places itself on the latter, it works independently of the class variable. Other characteristics of DI2 are: (1) static, where discretization of the variables takes place prior to an algorithm; (2) global, uses information about the variable as a whole to make the partitions and can still be applied with a scarce number of observations; (3) direct and splitting, splits the whole range of values into *k* intervals simultaneously; and (4) multivariate and univariate, DI2 can use either the whole dataset to create the intervals and discretize each variable or use each variable individually to create the respective intervals.

**Fig. 1:**
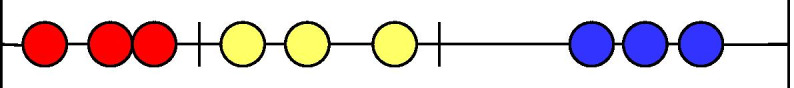
Illustration of equal-frequency method with 9 points along an axis and 3 categories. This method is based on the frequency of the items, where each category has the same number of items, in order to set the intervals

**Fig. 2:**
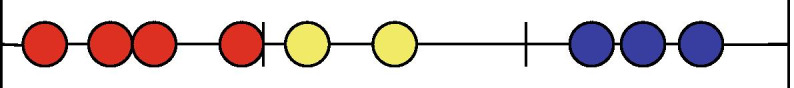
Illustration of equal-width method with 9 points along an axis and 3 categories. This method is based on the range taken by the items, where each category has the same width interval

**Fig. 3:**
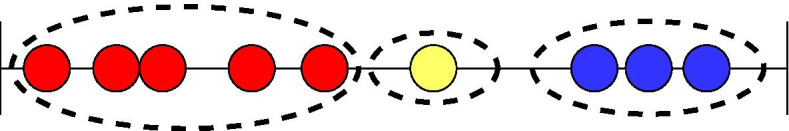
Illustration of K-means method with 9 points along an axis and 3 categories. This method is based in the k-means clustering, where each category is defined by a centroid

Some examples of unsupervised discretization methods are Proportional Discretization (PD), Fixed Frequency Discretization (FFD) [10], equal-width/frequency (also known as uniform and quantile) and k-means [11]. In this work, DI2 is compared with such classic discretization methods. These are illustrated in Figs. 1, 2, and 3.

### Normalization and feature scaling

While not mandatory, DI2 supports: *min-max scaling*,

1 $$\begin{aligned} X'= \frac{X - X_{min}}{X_{max} - X_{min}}, \end{aligned}$$

where *X* is an ordered set of observed values, and $X_{max}$ and $X_{min}$ are the maximum and minimum value within *X*; *z*-*score standardization* for normally distributed observations [12],

2 $$\begin{aligned} X'= \frac{X - {\overline{x}}}{S_n}, \end{aligned}$$

where *X* is an ordered set of observed values, ${\overline{x}}$ is the sample mean, and $S_n$ is the sample variance; and mean normalization,

3 $$\begin{aligned} X'= \frac{X - {\overline{x}}}{X_{max} - X_{min}}. \end{aligned}$$

where *X* is an ordered set of observed values, ${\overline{x}}$ is the sample mean, and $X_{max}$ and $X_{min}$ are the maximum and minimum value within *X*.

### Statistical hypotheses

In order to discretize the data into intervals, DI2 provides two statistical hypothesis tests: (1) ${\tilde{\chi }}^2$ test [13], and (2) Kolmogorov–Smirnov goodness-of-fit test [14].

In the aforementioned tests, the empirical distribution is matched with a theoretical continuous distribution^1^, provided by the SciPy open-source library [15], where the parameters are estimated through maximum likelihood estimation function. We consider the null hypothesis to be “the empirical probability distribution matches the theoretical probability distribution”. Considering a significance level of 0.05 and the number of degrees of freedom to be the number of categories inputted by the user minus one minus the number of estimated parameters [16] (excluding scale and location parameters). If the ${\tilde{\chi }}^2$ statistic is higher than the critical value at 0.05 we reject the hypothesis. The same logic is applied to the Kolmogorov–Smirnov statistic. The expected distribution of each category used in the ${\tilde{\chi }}^2$ test corresponds to the number of inputted categories by the user. The user can either choose the ${\tilde{\chi }}^2$ or the Kolmogorov–Smirnov goodness-of-fit as the *primary* fitting test. Both statistical tests yield properties of interest. While Kolmogorov–Smirnov does not provide an exhaustive characterization of the differences between the reference and empirical probability distributions as its statistic is derived from the highest distant point between the cumulative distributions, ${\tilde{\chi }}^2$ is dependent on the selected number of categories to assess the goodness of fitting. Having these concerns in mind, ${\tilde{\chi }}^2$ test is suggested as the default option unless a high number of data instances are available. In this latter case, the Kolmogorov–Smirnov test provides a finer-grained view as it more accurately models the empirical cumulative distribution.

DI2 informs the user of the selected distribution per column, the statistic of the applied test, and whether the computed statistic passes the goodness-of-fit test. One of the following scenarios can occur: (1) at least one theoretical distribution passes the statistical test, or (2) no theoretical distribution passes the statistical test. In both cases, the distribution with the lowest test statistic is chosen. The second scenario might be intentional. Consider the following, if the user knows that the empirical distribution is a sample from a population that follows a normal distribution, he can input the theoretical continuous distributions accordingly (normal distribution and its variants).

### Outlier correction

The Kolmogorov–Smirnov goodness-of-fit test can optionally be used to remove up to 5% outlier points, from the empirical distribution, according to the theoretical continuous distribution under assessment. Kolmogorov–Smirnov goodness-of-fit test returns a statistic (D statistic) measuring the maximum distance between the empirical and theoretical distributions,

4 $$\begin{aligned} D = \max \Big \{\max _{1 \le j \le n} \big \{\frac{j}{n} - F(X_j)\big \}, \max _{1 \le j \le n} \big \{F(X_j) - \frac{(j-1)}{n}\big \}\Big \}, \end{aligned}$$

where *n* is the number of observations, *j* is the index of a given observation, and *F* is the frequency of observation $X_j$. The first inner max function is referred as *D*-plus statistic, while the second inner max function is termed *D*-minus statistic. Using the *D* statistic we can pinpoint where the farthest point between the distributions is and remove it. After up to 5% of the observations have been removed, the iteration with the best Kolmogorov–Smirnov statistic is picked (from 0 outliers removed to up to 5%). The data produced by outlier removal is then used to run the main statistical hypothesis test picked (${\tilde{\chi }}^2$ or Kolmogorov–Smirnov). This correction guarantees the absence of penalizations caused by abrupt yet spurious deviations driven by the selected histogram granularity and help consolidate the choice of the theoretical continuous distribution. The outlier observations are only temporarily removed to fine tune the statistical hypothesis tests previously mentioned. Once the best fitting distribution is selected and category borders imputed, the library returns the original data (with all the outliers and missing values), not yielding impact on the remaining variables or subsequent data mining tasks.

### Multi-item discretization

After selecting the theoretical probability distribution that best fits the continuous variable, DI2 proceeds with the discretization. Given a desirable number of categories (bins), multiple cut-off points are generated using the inverse cumulative distribution function of the theoretical distribution. The cut-off points guarantee an approximately uniform frequency of observations per category, although empirical-theoretical distribution differences can underlie imbalances. The possibility to parameterize the number of bins is offered since in some application domains the desirable number is known a priori (e.g. well-defined number of gene activation levels for expression data analysis).

The optimal number of bins can be alternatively hyperparameterized. In supervised settings, cross-validation on training data can be pursued to this end. Similarly, in unsupervised settings, different cardinalities can be assessed against a well-defined quality criteria (e.g. silhouette in clustering solutions or number of statistically significant patterns in biclustering solutions) to estimate the number of bins. Alternatives for parameterizing the number of bins, including heuristic searches have been suggested [17]. In clinical domains, Maslove et al. [18] used an heuristic for determining the number of bins when discretizing data with unsupervised methods.

**Fig. 4:**
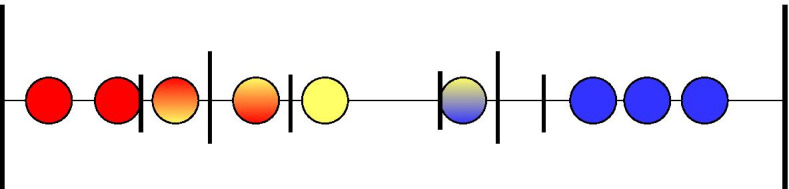
Illustration example of discretization with 9 points along an axis and 3 categories considering border values (values which belong to 2 categories)

Unlike other well-known unsupervised discretization methods,(e.g. the aforementioned methods) DI2 supports multi-item assignments by identifying border values for each category, this is exemplified in Figure 4. Note also that in the presence of algorithms able to handle multi-items derived from category borders, the items-boundary problem associated with different bin choices is ameliorated. To this end, the user can optionally also define a boundary proximity percentage (between 0 and 50%, 20% being the default) to affect the distance from category borders. Let us introduce an example: the discretization of a variable following a Normal distribution, N(0, 1), with three categories. The cut-off points are − 0.43 and 0.43. To allow the presence of border values, observations with values near the frontiers of discretization are assigned with two categories. By default, a proximity of 20% to a discretization boundary is assumed for the assignment of multiple items. Proximity percentage is estimated by dividing the area under the probability distribution curve between the observation and the closest discretization boundary by the area between the discretization boundaries of the observation’s category. In the given example, observations falling between − 0.63 and − 0.43, as well as between − 0.43 and − 0.26, are assigned with two items. It can also be observed that the proximity percentages translate into border boundaries (smaller brackets) being placed to the left and right of the discretization boundary (medium-sized brackets).

## Implementation

**Fig. 5:**
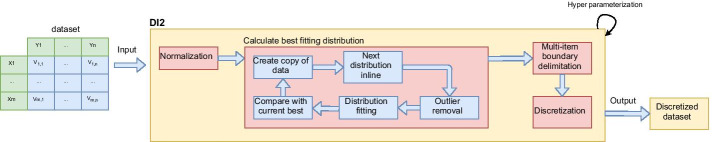
The flowchart of DI2. From data input, passing through data normalization, fitting of categories, and finally discretization

DI2 tool is fully implemented in Python 3.7^2^ (Additional file 1). DI2 is provided as an open-source method at GitHub with well-annotated APIs and notebook tutorials for a practical illustration of its major functionalities. The algorithm workflow is shown in Algorithm 1 and the Kolmogorov–Smirnov correction is shown in Algorithm 2. DI2 workflow is further shown in Figure 5. All the code was executed on a computer with Intel(R) Core(TM) i5-8265U CPU @ 1.60 GHz 1.80 GHz, and 24 GB of RAM.

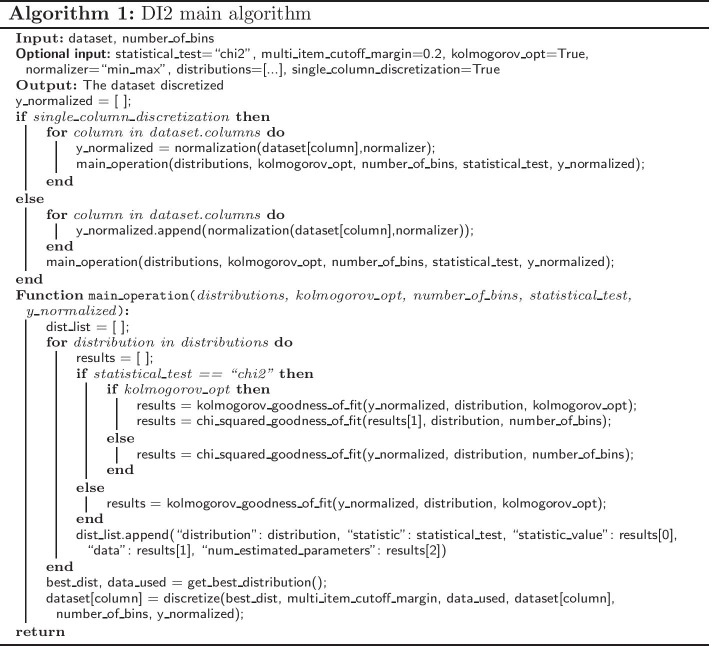

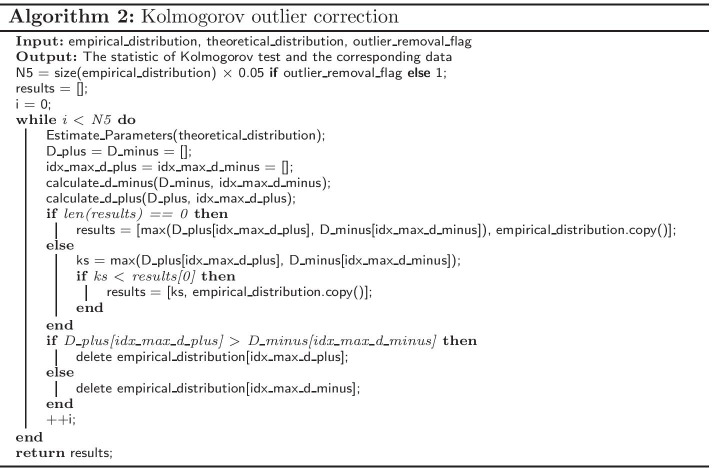





## Results and discussion

In order to illustrate some of the DI2 properties, we considered two published datasets: (1) the *breast-tissue* *dataset* [19], containing electrical impedance measurements in samples of freshly excised tissue from the breast, and (2) the *yeast* *dataset* [20], containing molecular statistics variables. Both of these are available at the UCI Machine Learning repository [21] and a more detailed variable explanation is presented in Tables 1 and 2.

**Table 1:** Variables of the *breast-tissue* dataset and their respective description

Variables	Type	Description
I0	Continuous	Impedivity (ohm) at zero frequency
PA500	Continuous	Phase angle at 500 KHz
HFS	Continuous	High-frequency slope of phase angle
DA	Continuous	Impedance distance between spectral ends
Area	Continuous	Area under spectrum
A/DA	Continuous	Area normalized by DA
Max IP	Continuous	IP maximum of the spectrum
DR	Continuous	Distance between I0 and real part of the maximum frequency point
P	Continuous	Length of the spectral curve
Class	Categorical	Carcinoma, fibro-adenoma, mastopathy, glandular, connective, adipose

**Table 2:** Variables of the *yeast* dataset and their respective description

Variables	Type	Description
Sequence	Text	Accession number
mcg	Continuous	McGeoch’s method for signal sequence recognition
gvh	Continuous	von Heijne’s method for signal sequence recognition
alm	Continuous	Score of the ALOM membrane spanning region prediction program
mit	Continuous	Discriminant score of amino acid content of N-terminal regions
erl	Binary	Presence of retention signals in the endoplasmic reticulum lumen
pox	Continuous	Peroxisomal targeting signal in the C-terminus
vac	Continuous	Discriminant score of aminoacid content of vacuolar/extracellular proteins
nuc	Continuous	Discriminant score of nuclear localization signals
Class	Categorical	Localization site of protein.

DI2 is executed with ${\tilde{\chi }}^2$ as the main statistical test, with and without Kolmogorov outlier removal, with single and whole column discretization, and 3, 5 and 7 categories per variable outputted. Predictive performance is further assessed against raw continuous data. The acronyms for the probability distributions referred throughout this section are described in Table 3.

**Table 3:** Theoretical probability distribution acronyms (for full list visit https://docs.scipy.org/doc/scipy/reference/stats.html—SciPy statistical functions)

Distribution acronym	Description
Alpha	Alpha continuous random variable
Exponnorm	Exponentially modified Normal continuous random variable
Foldcauchy	Folded Cauchy continuous random variable
Recipinvgauss	Reciprocal inverse Gaussian continuous random variable
Frechet_r	Frechet right (or Weibull minimum) continuous random variable
Mielke	Mielke Beta-Kappa / Dagum continuous random variable
Johnsonsu	Johnson SU continuous random variable
Johnsonsb	Johnson SB continuous random variable
Genextreme	Generalized extreme value continuous random variable
chi2	Chi-squared continuous random variable
genlogistic	Generalized logistic continuous random variable
Laplace	Laplace continuous random variable
Genhalflogistic	Generalized half-logistic continuous random variable
Gengamma	Generalized gamma continuous random variable
Pearson3	Pearson type III continuous random variable

### Case study: *breast-tissue dataset*

**Fig. 6:**
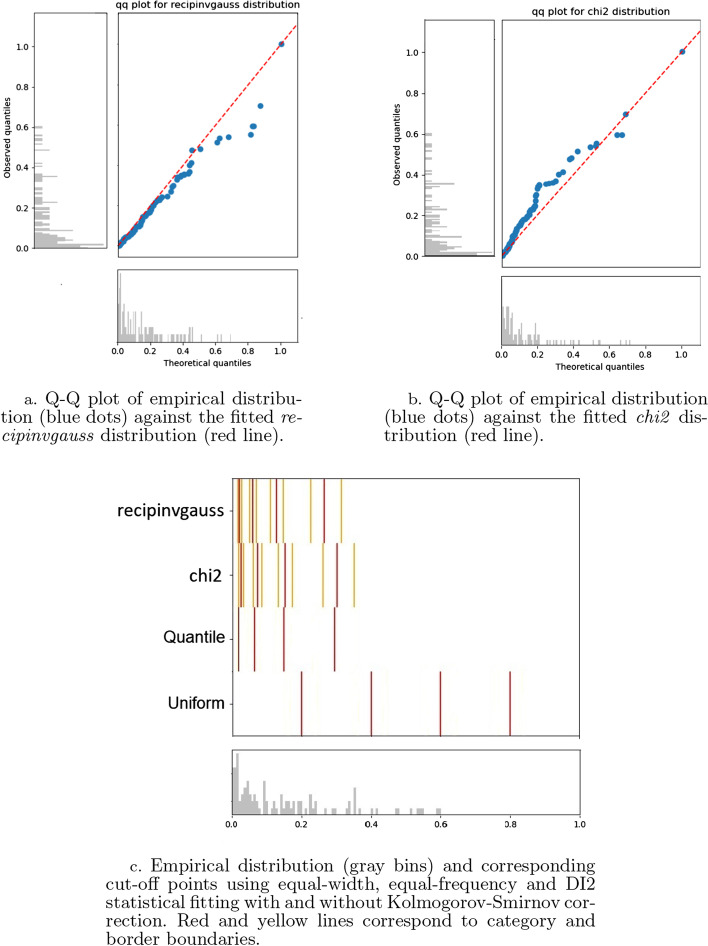
Distribution matching of DA variable from breast-tissue againt two statistical distributions (*recipinvgauss* in **a** and *chi2* in **b**, as well as the corresponding discretization boundaries and border values in V.c

The *breast-tissue* dataset contains 106 data instances and 10 variables (9 continuous and 1 categorical), presented in Table 1. The gathered results show the decisions placed by DI2 in the absence and presence of Kolmogorov–Smirnov optimization.

Table 4 shows the distributions yielding best fit for each continuous variable of the dataset. Variables “I0”, “PA500”, “A/DA”, “DR”, and “P” remained unchanged with a removal of up to 5% of outlier points. Variables “HFS” and “Area” produced better results in the ${\tilde{\chi }}^2$ test with the removal of outliers solidifying the distribution choice. Finally, the fitting choice changed for variables “DA” and “Max IP” under the ${\tilde{\chi }}^2$ test, revealing a more solid choice from the analysis of the residuals.

**Table 4:** Best fitting distributions for each continuous variable, without and with Kolmogorov–Smirnov correction

Variables	Without opt.	${\tilde{\chi }}^2$ statistic	*p*-value >0.05 (${\tilde{\chi }}^2$)	D statistic	With opt.	${\tilde{\chi }}^2$ statistic	*p*-value >0.05 (${\tilde{\chi }}^2$)	D statistic
I0	alpha	8.8	False	0.12	alpha	8.8	False	0.11
PA500	exponnorm	2.98	True	0.07	exponnorm	2.98	True	0.07
HFS	foldcauchy	2.25	True	0.07	foldcauchy	1.57	True	0.07
DA	recipinvgauss	1.6	True	0.06	chi2	1.01	True	0.06
Area	frechet_r	0.5	True	0.07	frechet_r	0.25	True	0.05
A/DA	mielke	1.17	True	0.06	mielke	1.17	True	0.05
Max IP	johnsonsu	4.72	True	0.05	alpha	1.09	True	0.07
DR	johnsonsb	1.2	True	0.05	johnsonsb	1.2	True	0.05
P	genextreme	5.13	True	0.09	genextreme	5.13	True	0.09

Both ${\tilde{\chi }}^2$ (primary) and KS statistics are shown

Considering “DA” variable, Fig. 6a, b show its Q-Q (quantile-quantile) plot, offering a view on the adequacy of the statistical fitting. In this context, we depict histograms for the empirical data with 100 bins (blue dots), to better visualize the impact of outlier removal, and the best theoretical distribution picked without and with Kolmogorov–Smirnov correction (red line). A moderate improvement from Fig. 6a, b can be detected, with the empirical quantiles (blue dots) being closer to the theoretical continuous quantiles (red line).

After the fitting stage, cut-off points are calculated to produce the final categories. Figure 5c compares different discretization options: quantile, uniform, and the two best fitting theoretical continuous distributions (without and with Kolmogorov–Smirnov optimization). Category cut-off points are marked as red lines, and the border values cut-off points in yellow. This analysis shows how critical discretization can be for determining the inclusion or exclusion of high density bins. The ability of DI2 to assign multiple items using borders can thus be explored by symbolic approaches to mitigate vulnerabilities inherent to the discretization process [22, 23].

### Case study: *yeast dataset*

The *yeast* dataset contains 1484 data instances and 10 variables, including the sample identification, class, and 8 molecular statistics variables (Table 2). In the previous analysis, *breast-tissue dataset* was considered to compared DI2 category cut-off points against alternative unsupervised discretization procedures – quantile (equal-frequency) and uniform (equal-width). The *yeast* data is used to comprehensively assess the predictive capabilities of discretization approaches, including the k-means method.

Table 5 displays the results of the statistical tests produced by DI2 when applied to each variable independently and the whole dataset together, considering 5 categories per variable. As presented in Table 5, the empirical distribution of a variable does not always match a known theoretical distribution with statistical significance (e.g. variable “alm”). Nonetheless, the theoretical distribution with the lowest test statistic is still selected in an effort to ameliorate bad discretization decisions by preventing critically misadjusted probability distributions.

**Fig. 7:**
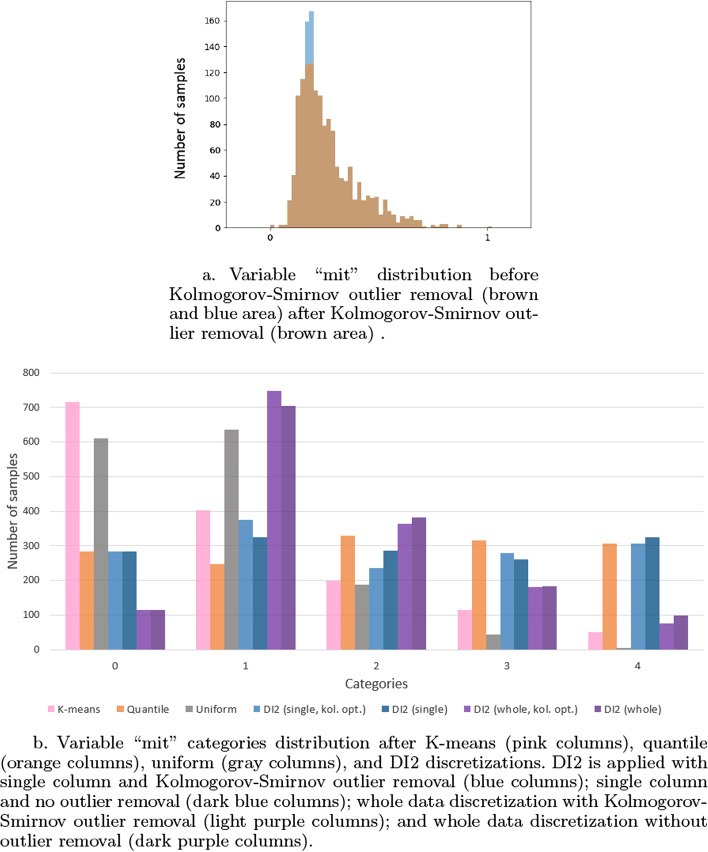
Variable “mit” distribution (**a**). Categories distribution after k-means, quantile, uniform, and DI2 discretization (**b**)

**Fig. 8:**
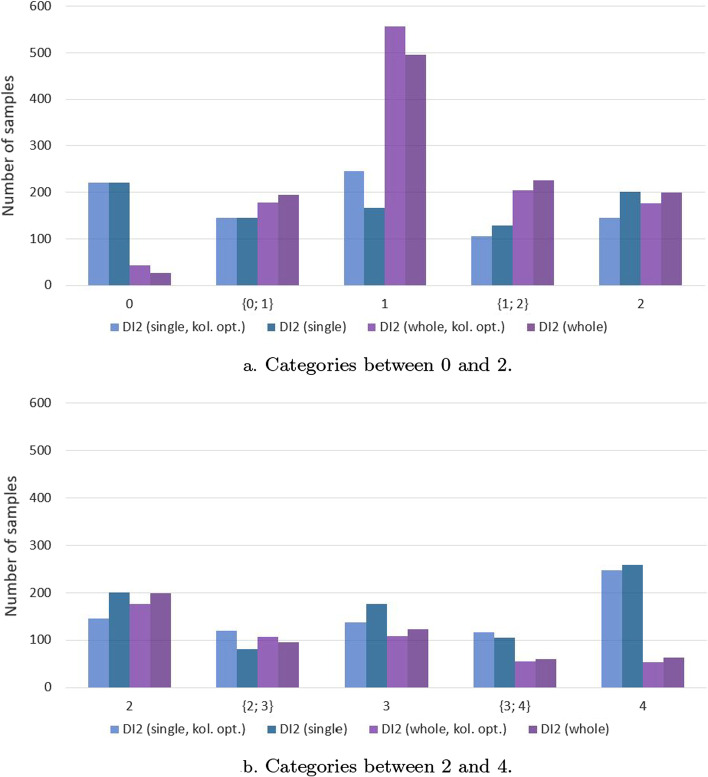
Variable “mit” categories distribution after DI2 discretization with different settings with border values. Single column discretization with Kolmogorov–Smirnov outlier removal (light blue columns), single column discretization without Kolmogorov–Smirnov outlier removal (dark blue columns), whole dataset discretization with Kolmogorov–Smirnov outlier removal (light purple columns), whole discretization without Kolmogorov–Smirnov outlier removal (dark purple columns)

Figure 7a displays the distribution of values in the variable “mit” before outlier removal (brown and blue area of histogram) and after outlier removal (brown area of histogram). Figure 7b compares the distribution of the categories of all the discretization techniques (DI2, quantile, uniform, and k-means), and further assesses the impact of outlier removal had in categorizing the data in different executions of DI2. Figure 8 presents the frequency distribution of observation per category, as well as intermediate categories produced by DI2’s border values.

**Table 5:** Best fitting distributions for each continuous variable, without and with Kolmogorov–Smirnov outlier removal, considering 5 categories per variable

Variables	Without opt.	${\tilde{\chi }}^2$ statistic	*p*-value >0.05 (${\tilde{\chi }}^2$)	D statistic	With opt.	${\tilde{\chi }}^2$ statistic	*p*-value >0.05 (${\tilde{\chi }}^2$)	D statistic
mcg	foldcauchy	3.72	True	0.08	exponnorm	3.18	True	0.02
gvh	genlogistic	3.57	True	0.03	genlogistic	2.02	True	0.02
alm	genlogistic	17.00	False	0.05	genlogistic	12.08	False	0.03
mit	exponnorm	19.23	False	0.05	exponnorm	6.11	True	0.03
pox	chi2	$4.4 \times 10^{-14}$	True	0.99	gengamma	$4.2 \times 10^{-14}$	True	0.99
vac	laplace	20.99	False	0.08	pearson3	14.18	False	1.00
nuc	exponnorm	1116.63	False	0.26	mielke	795.28	False	0.26
all variables	genhalflogistic	45.69	False	0.25	genhalflogistic	10.25	False	0.21

The performed analysis for the *yeast dataset* shows how critical the category border, previously discussed in more detail with the *breast-tissue* dataset, can be. The ability of DI2 to assign multiple items using borders can be explored by symbolic approaches to mitigate vulnerabilities inherent to the discretization process as discussed in the following subsection.

### Predictive performance

To assess the predictive impact of DI2, we reuse the *yeast* dataset, applying a cross-validation scheme with 10 folds, and six supervised classification methods: Naive Bayes [24], Random Forest [25], support vector machines using Sequential Minimal Optimization (SMO) [26], C4.5 [27], Multinomial Logistic Regression Model (MLRM) [28] and FleBiC [29]. Discretization procedures are applied with 3, 5 and 7 categories per variable. To preserve the soundness of assessments, the discretization thresholds are learned only on the training data per fold. The testing data instances are then discretized using the learned discretization thresholds from training data.

**Fig. 9:**
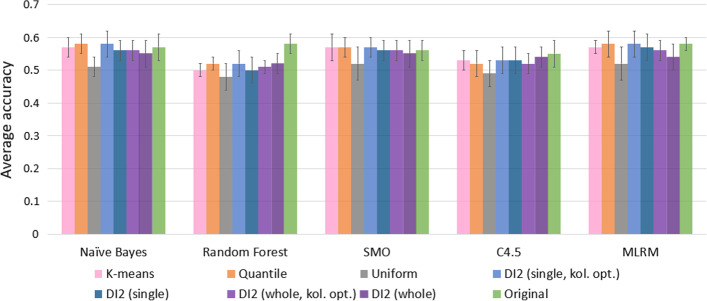
Average accuracy per classifier and discretization method available without border values and considering 5 categories per variable﻿(for more information consult Additional file 2). From left to right in each group of bars: K-means, Quartile, Uniform, DI2 (single, kol. correction), DI2 (single), DI2 (whole, kol. correction), DI2 (whole) and original data

Figure 9 presents the results of the aforementioned models with the original numerical data and a discretization of 5 categories per variable. In each model, DI2, with configurations of single column discretization and outlier removal, is among the top performing procedure. In particular, the C4.5 model, DI2, with configurations of combined column discretization, achieved the highest accuracy compared with other discretization methods. Considering Naïve Bayes and SMO models, DI2 achieves competitive performance against the original numerical data, with a generally higher average accuracy for single column discretizations, yet not yielding statistically significant improvements.

**Fig. 10:**
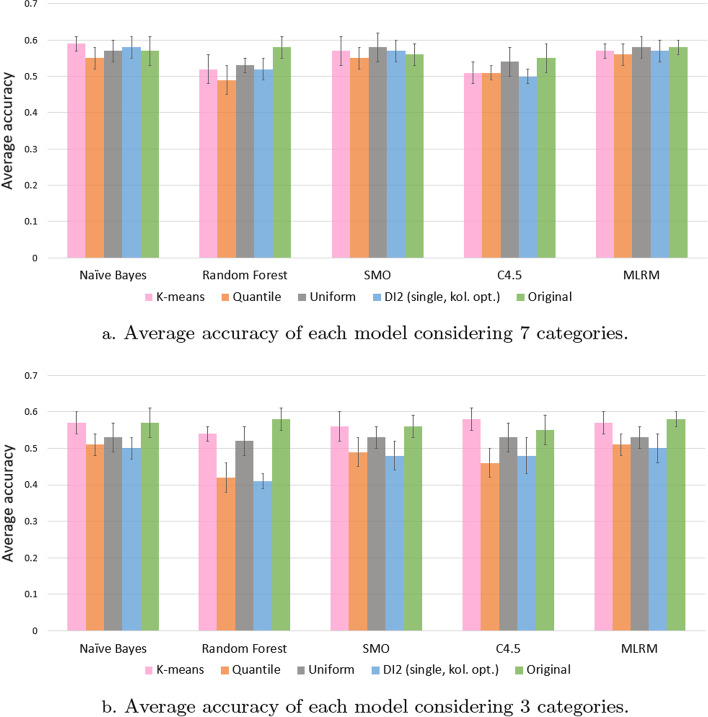
Accuracy when executing different models with multiple discretization methods. From left to right the bars are: K-means, Quartile, Uniform, DI2 (single, kol. correction) without border values and original data

Figure 10 displays the average accuracy achieved by each model with a discretization of 3 and 7 categories per variable. Results considering 3 and 7 categories were not as optimal as with 5 categories, in terms of accuracy. Nonetheless, these results further encourage hyperparameterization to find an optimal number of bins.

**Fig. 11:**
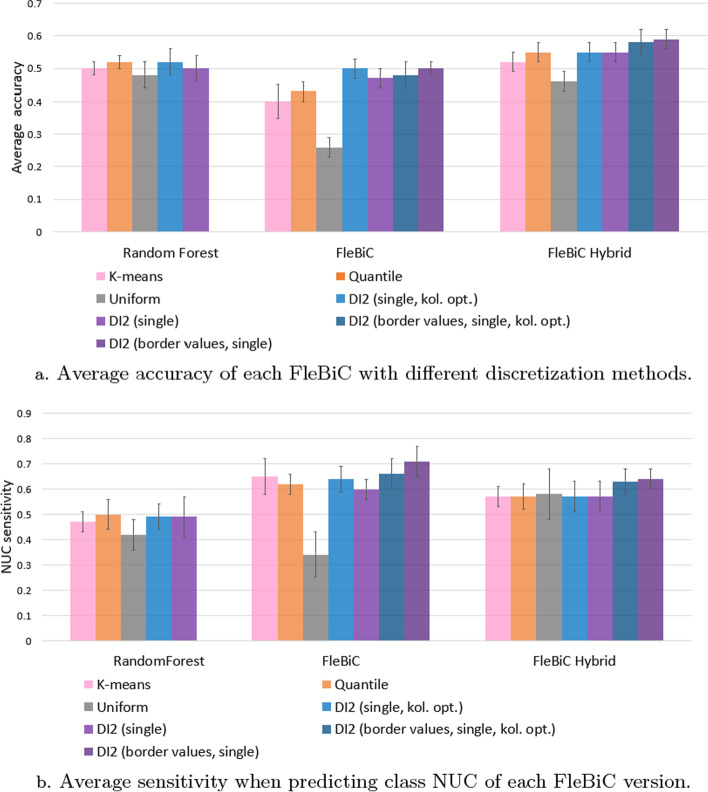
Accuracy when executing different FleBiC versions, and Sensitivity of when predicting class NUC, with multiple discretization methods considering 5 categories per variable (for more information consult Additional file 2). From left to right the bars are: K-means, Quartile, Uniform, DI2 (single, kol. correction), DI2 (single), DI2 (border values, single, kol. correction) and DI2 (border values, single)

**Fig. 12:**
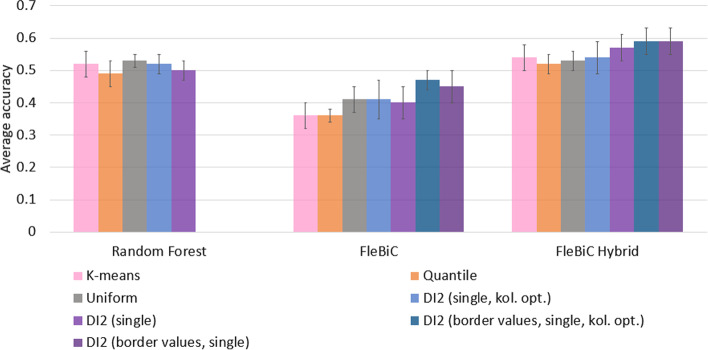
Accuracy when executing different FleBiC versions with multiple discretization methods considering 7 categories per variable. From left to right the bars are: original data, K-means, Quantile, Uniform, DI2 (single, kol. correction), DI2 (single), DI2 (border values, single, kol. correction), DI2 (border values, single)

In order to fully test out the potential of DI2, we now considered border values. FleBiC [29] is a classifier able to place decisions based on multi-item assignments. Other approaches, such as BicPAMS [4] (a patterned-based biclustering algorithm), can be alternatively consider to accommodate border values and thus minimize potential discretization drawbacks. FleBiC is here executed as a stand-alone classifier and as an adjunct classifier to guide decisions of Random Forests, where decisions are derived from both the probabilistic outputs of FleBiC (50%) and Random Forests (50%), which will be denoted by FleBiC Hybrid. Figure 11 shows the results of FleBiC and FleBiC Hybrid. In terms of average accuracy (Figure 11.a), both FleBiC and FleBiC Hybrid yield higher predictive accuracy with DI2 method than with other discretization methods. Within the different settings of DI2, the best predictive accuracy is achieved for FleBiC Hybrid when the predictive model considers border values. Figure 12 presents the results when considering 3 and 7 categories. Finally, when considering the sensitivity of the NUC outcome (Figure 11.b), we can see that the incorporation of border values plays a decisive role, making it possible to break through a ceiling on the NUC predictability against discretization methods unable to consider border values. More details on the relevance of border values to improve the sensitivity of other classes are provided in supplementary material. This analysis shows that the use of border values can yield significant improvements.

To assess if the previous differences in predictive accuracy are statistically significant, a one-tailed paired *t*-test is applied. We consider the alternative hypothesis (*p*-value < 0.05) to be “DI2 is superior to the identified discretization procedure using the same classifier”. Results obtained considering the discretization of 5 categories per variable are presented in Table 6. DI2 shows statistically significant improvements against uniform discretization in all classification models. DI2, with single column and optimized single column configurations, despite displaying competitive predictive accuracy in most of the classifiers against k-means and quantile discretizations, it does not show statistically significant improvement. However, when considering FleBiC, DI2 outperformed all remaining discretization methods, with or without border values (*p*-value<0.05). In FleBiC Hybrid, DI2 also outperformed all other discretization methods with the exception of quantile discretization when no border values are considered.

**Table 6:** Gathered *p*-values from statistically testing the superiority of DI2 with respect to predictive accuracy against alternative discretization procedures, and original data, using one-tailed paired *t*-test and considering 5 categories per variable (complementary information in Additional file ﻿3﻿)

	DI2 (single)				DI2 (single, optimized)			
	K-means	Quantile	Uniform	Original	K-means	Quantile	Uniform	Original
Naïve Bayes	0.686	0.897	**0**.**005**	0.719	0.287	0.431	**0**.**002**	0.325
Random Forest	0.404	0.921	0.101	0.998	0.126	0.653	**0**.**016**	0.998
SMO	0.980	0.968	**0**.**014**	0.456	0.790	0.773	**0**.**017**	0.441
C4.5	0.500	0.345	**0**.**044**	0.965	0.230	0.194	**0**.**013**	0.891
MLRM	0.500	0.907	**0**.**009**	0.803	0.316	0.821	**0**.**013**	0.588
FleBiC	**0**.**001**	**0**.**007**	**1.9E−08**	–	**2.1E−05**	**1.0E−04**	**6.7E−09**	–
FleBiC Hybrid	**5.4E−04**	0.693	**5.2E−05**	–	**0**.**030**	0.873	**2.0E−04**	–

	DI2 (whole)				DI2 (whole, optimized)			
	K-means	Quantile	Uniform	Original	K-means	Quantile	Uniform	Original
Naïve Bayes	0.948	0.991	**0**.**020**	0.965	0.662	0.822	**0**.**004**	0.712
Random Forest	0.066	0.426	**0**.**012**	0.992	0.074	0.666	0.195	0.999
SMO	0.906	0.914	**0**.**042**	0.641	0.805	0.813	**0**.**026**	0.406
C4.5	0.085	0.072	**0**.**004**	0.702	0.687	0.500	**0**.**028**	0.958
MLRM	0.952	0.986	0.148	0.993	0.721	0.896	**0**.**047**	0.942

	DI2 (borders, single)				DI2 (borders, single, optimized)			
	K-means	Quantile	Uniform	Original	K-means	Quantile	Uniform	Original
FleBiC	**8.0E−05**	**7.3E−05**	**1.5E−08**	–	**0**.**002**	**0**.**016**	**9.1E−08**	–
FleBiC Hybrid	**1.4E−05**	**0**.**001**	**4.3E−06**	–	**6.1E−04**	0.084	**1.0E−04**	–

DI2 is assessed without and with border values, single column and whole dataset, and in the absence and presence of outlier removal

Bold values indicate that the accuracy achieved using DI2 discretization is statistically superior against the corresponding discretization

The benefits of discretization go beyond the previously assessed predictive settings. In the context of deep learning approaches, Rabanser et al. [30] surveyed the effect of data input and output transformations on the predictive performance of several neural forecasting architectures, concluding that the WaveNet model, when input data is discretized, yields best results.

### Scalability

**Fig. 13:**
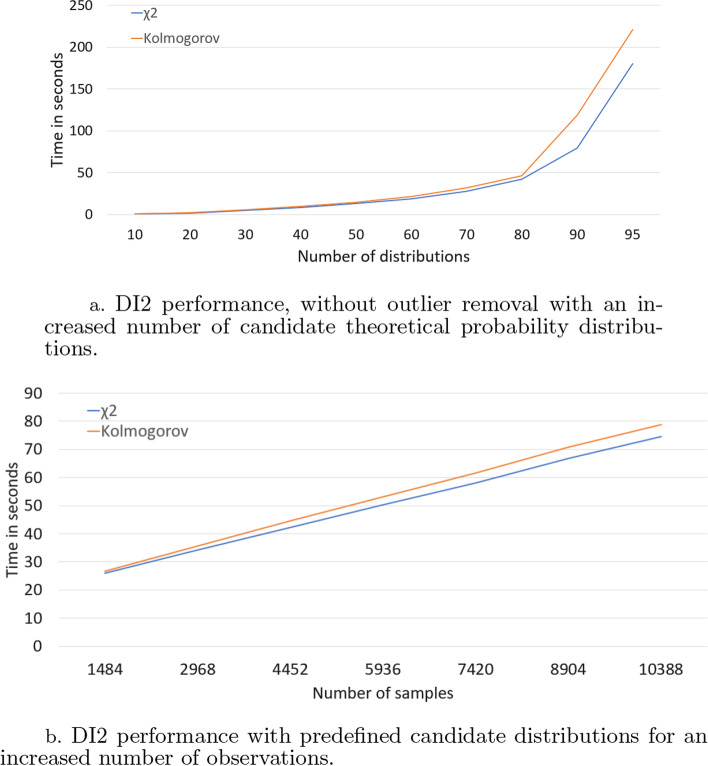
Computational time efficiency of DI2 (without outlier removal) according to the number of underlying probability distributions (**a**) and number of observations. Candidate distributions (from 0 to 95) are added with respect to ascending computational time, i.e. from fastest to slowest estimation of the theoretical distribution’s parameters

The execution time of DI2 is presented in Fig. 13. Figure 13a displays the efficiency according to the number of tested theoretical distributions (from fastest to slowest in terms of parameter estimation) using the *yeast* dataset (1484 observations). Figure 13.b depicts how the computational time varies in accordance with the number of observations for the DI2 default setting, considering the *yeast* data with all variables.

## Conclusion

This work proposed a new unsupervised method for data discretization, DI2, that takes into account the underlying data regularities, the presence of outlier values disrupting expected regularities, as well as the relevance of border values. A tool for the autonomous, prior-free discretization of biological data with arbitrarily skewed variable distributions is provided to this end.

Our study showed that DI2 is a viable and robust discretization procedure when compared against well-established unsupervised discretization methods. Statistical tests applied to assess differences in performance confirm that DI2 generally outperforms alternative discretization methods with statistical significance. The combined use of DI2 within classification tasks results in either competitive or superior levels of predictive accuracy. DI2 as the unique feature of allowing the incorporation of border values. FleBiC, a classifier able to accommodate border values, achieved statistically significant performance improvements in the presence of multi-item assignments.

## Availability and requirements

Project name: DI2: prior-free and multi-item discretization.

Software homepage: https://github.com/JupitersMight/DI2.

Programming language: Python.

Other requirements: python 3.7, pandas 1.2.4, scipy 1.5.1 and numpy 1.20.2.

License: MIT License.

Any restrictions to use by non-academics: None.

## Supplementary information

**Additional file 1.** Folder containing DI2 and an example in Jupyter Notebook using Breast Tissue dataset example.

**Additional file 2.** File with the average accuracy achieved by models with discretization method considering 5 categories.

**Additional file 3.** File with the accuracy achieved in cross validation by each discretization method in each model considering 5 categories.

## Data Availability

The software is available at https://github.com/JupitersMight/DI2. The data is publicly available at the UCI Machine Learning repository [31]. The *breast-tissue* dataset is available at: https://archive.ics.uci.edu/ml/datasets/Breast+Tissue and the *yeast* dataset is available at: https://archive.ics.uci.edu/ml/datasets/yeast.

## Abbreviations

DI2: Distribution Discretizer
Quantile: Equal-frequency
Uniform: Equal-width
Q-Q plot: Quantile–Quantile plot
FleBiC: Flexible Biclustering-based Classifier
BicPAMS: Biclustering based on PAttern Mining Software

## Footnotes

1 https://docs.scipy.org/doc/scipy/reference/stats.html.

2 DI2 currently uses the following libraries: pandas 1.2.4, scipy 1.5.1, and numpy 1.20.2
